# Low expression of *WWC1*, a tumor suppressor gene, is associated with aggressive breast cancer and poor survival outcome

**DOI:** 10.1002/2211-5463.12659

**Published:** 2019-06-01

**Authors:** Zhanwei Wang, Dionyssios Katsaros, Nicoletta Biglia, Yi Shen, Yuanyuan Fu, Maarit Tiirikainen, Herbert Yu

**Affiliations:** ^1^ University of Hawaii Cancer Center Honolulu HI USA; ^2^ Department of Surgical Sciences Gynecology AOU Città della Salute University of Turin Italy; ^3^ Department of Surgical Science Division of Obstetrics and Gynecology Mauriziano Hospital University of Torino School of Medicine Turin Italy; ^4^ Department of Molecular Biosciences and Bioengineering University of Hawaii at Manoa Honolulu HI USA

**Keywords:** breast cancer, methylation, overall survival, relapse‐free survival, *WWC1*

## Abstract

The WW and C2 domain containing 1 (WWC1) gene encodes a protein named WWC1 (or KIBRA), which is involved in the Hippo signaling pathway. *WWC1* is often lost in triple‐negative breast cancer and has been shown to suppress tumor metastasis. In this study, 470 breast cancer patients were recruited and *WWC1* expression in the tumor samples was measured with quantitative reverse transcriptase PCR. Associations of *WWC1* expression with breast cancer survival were analyzed using the Cox proportional hazards regression model and Kaplan–Meier survival analysis. The relationship between *WWC1* expression and methylation was evaluated in a dataset from The Cancer Genome Atlas. Using our microarray data on gene expression and the Ingenuity Pathway Analysis, we predicted the *WWC1*‐associated signaling pathways in breast cancer. Our results showed that low expression of *WWC1* was significantly associated with advanced‐stage diseases, high‐grade tumors, and estrogen receptor‐ or progesterone receptor‐negative status. Compared to those with high expression, patients with low *WWC1* had higher risk of breast cancer relapse [hazard ratio (HR) = 2.06, 95% confidence interval (CI): 1.26–3.37] and higher risk of death (HR = 2.76, 95% CI: 1.51–5.03). The association with relapse‐free survival remained significant after adjustment for disease stage, tumor grade, and hormone receptor status and was replicated in a public dataset. Analysis of high‐throughput gene expression data indicated that *WWC1* was involved in the Hippo signaling pathway. Online data also suggested that DNA methylation was inversely associated with *WWC1* expression. The study confirmed that low *WWC1* expression was associated with aggressive breast cancer and poor survival outcomes.

**Abbreviations**
EMT: epithelial‐to‐mesenchymal transition
ER: estrogen receptor
HR: hazard ratio
IPA: Ingenuity Pathway Analysis
OS: overall survival
PR: progesterone receptor
qRT‐PCR: quantitative reverse transcriptase PCR
RFS: relapse‐free survival
TCGA: The Cancer Genome Atlas
TNBC: triple‐negative breast cancer
WWC1: WW and C2 domain containing 1

Breast cancer is the most common malignancy and a leading cause of cancer death among women worldwide. The malignancy accounts for 25% of all cancer cases and 15% of all cancer deaths. In developed countries, these figures are even higher, about 50% of all cancer cases and 38% of cancer deaths 1. Breast cancer is a heterogeneous disease 2, and accurately characterizing breast tumor at molecular and genetic levels is important in identifying sensitive and responsive targets for treatment. Triple‐negative breast cancer (TNBC) is a group of aggressive tumors with poor prognosis. Nearly 70% of TNBC had a deletion in chromosome 5 (5q11–5q35) 3, 4, 5. A recent study by Knight *et al*. indicated that a gene in the region named the WW and C2 domain containing 1 (WWC1) gene could inhibit breast cancer progression. The gene encodes a protein called WWC1 (or KIBRA) which interacts with PTPN14 (protein tyrosine phosphatase nonreceptor 14) and inhibits the oncogenic activity of YAP/TAZ 6.

WW and C2 domain containing 1 consists of two WW domains in the amino‐terminus, an internal C2‐like domain, and a carboxy‐terminal glutamic acid‐rich stretch. The protein was originally found to be predominantly expressed in the kidneys and brain 7 and is suspected to be involved in memory performance and associated with age‐dependent risk of Alzheimer's disease 8, 9, 10, 11. Studies also found that the protein was involved in regulation of cell migration 12, 13, 14. Genetic screening of Drosophila suggests that this gene may function as a potential tumor suppressor by regulating the Hippo signaling pathway 15, 16, 17 which controls tissue growth and tumorigenesis, inhibits cell proliferation, and promotes apoptosis 18, 19, 20, 21, 22, 23. *WWC1* associates with both large tumor suppressor (Lats) 1 and Lats2 to regulate the Hippo signaling in human cells 24. One study indicated that activation of the Hippo signaling pathway by WWC1 in breast cancer cells could suppress epithelial‐to‐mesenchymal transition (EMT) 25. Another showed that WWC1 regulated epithelial cell polarity by suppressing apical exocytosis through direct inhibition of aPKC kinase activity in the PAR3/PAR6/aPKC complex 26. Loss of epithelial cell polarity is intricately related to tumor progression and invasion 27. A previous study also found that WWC1 formed a complex with dynein light chain 1, an estrogen receptor‐α (ER)‐interacting protein, and played a role in ER transactivation in breast cancer cells 24.

Despite the growing evidence on WWC1's involvement in breast cancer progression, no study has evaluated its relationship with tumor characteristics and patient survival. To address these issues, we conducted a clinical study to examine *WWC1* expression in association with patient clinical and pathological features as well as disease outcomes.

## Materials and methods

### Patients and tumor samples

Breast tumor samples were collected from patients during surgery in a university hospital between January 1998 and July 1999 and in Mauriziano Hospital between October 1996 and August 2012. Both hospitals are affiliated with University of Turin. The patients were followed regularly after surgery for postoperative treatment and checkup. The average age of patients at surgery was 58 years (range: 23–89). The median relapse‐free survival (RFS) and overall survival (OS) were 82.2 months (range: 1.7–196.4) and 83.6 months (range: 4.2–196.4), respectively. Information on disease stage, tumor grade, hormone receptor status, follow‐up time, and survival outcomes was extracted from patient medical records and follow‐up contacts. A detailed description of the information has been described elsewhere 28. The study was approved by the ethical review committees in the hospitals, and informed written consents were obtained from the patients. The study methodologies conformed to the standards set by the Declaration of Helsinki.

### RNA extraction

Total RNA was extracted from 470 fresh‐frozen breast tumor samples using the AllPrep DNA/RNA Kit (Qiagen, Hilden, Germany). The concentrations and qualities of RNA were measured with the NanoDrop 1000 spectrophotometer, and the specimens were stored at −80 °C until analysis. The RNA integrity was assessed on the Agilent Bioanalyzer (Agilent Technologies, Santa Clara, CA, USA).

### Quantitative reverse transcriptase PCR

*WWC1* expression was analyzed in 470 tumor samples using quantitative reverse transcriptase PCR (qRT‐PCR). PCR primers were designed and synthesized by IDT (San Diego, CA, USA). Total RNA (1 μg) was reverse‐transcribed using the cDNA Reverse Transcription Kit (LifeTech, Carlsbad, CA, USA); qRT‐PCR was performed with the SYBR Select Master Mix (LifeTech) in a 384‐well plate using the Roche LightCycler 480 real‐time PCR detection system (Roche Diagnostics Ltd., Rotkreuz, Switzerland). In each PCR (10 μL), cDNA template (0.5 μL) was mixed with 200 nm primers and 5 μL SYBR PCR master mix. Cycling conditions included the following: 50 °C for 2 min to activate UDG, 95 °C for 2 min to activate Taq polymerase, 40 cycles of 95 °C for 15 s and 60 °C for 60 s, and 60 °C for 1 min. The cycle threshold (Ct) was determined for each sample in triplicate. *WWC1* expression was normalized to *GAPDH*, and the relative value was calculated as expression index (EI), using the formula 1000 × 2^(−ΔCt)^, where ΔCt = Ct(*WWC1*) − Ct(*GAPDH*). PCR primers were 5′‐TCCGCAGTCCTGGAAACATT‐3′ (forward) and 5′‐GTGGATTCCCAATGAGCCGA‐3′ (reverse) for *WWC1*, and 5′‐GTCAAGGCTGAGAACGGGAA‐3′ (forward) and 5′‐AAATGAGCCCCAGCCTTCTC‐3′ (reverse) for *GAPDH*.

### Breast cancer microarray analysis

Of the 470 tumor samples analyzed by qRT‐PCR for *WWC1* expression, 204 samples were also used for microarray analysis with the Illumina Expression BeadChip (HumanRef‐8 v3) (Illumina, San Diego, CA, USA) according to the manufacturer's protocol, which was described elsewhere 29, 30. In brief, microarray data were preprocessed by the BeadStudio software and analyzed using the R statistical software and Bioconductor 31. The Lumi R package was used to normalize the data 32. The genefilter package was applied for nonspecific filtering; gene expression levels with interquartile range < 0.5 were discarded.

### Statistical methods

Values of *WWC1* EI were categorized into three groups according to the tertile distribution: low, medium, and high. Chi‐square test was performed to evaluate the association between *WWC1* expression and clinicopathologic characteristics of breast cancer patients. The association of *WWC1* expression with breast cancer survival was analyzed using the Kaplan–Meier method and log‐rank test. Univariate and multivariate Cox proportional hazards regression models were used to determine the hazard ratio (HR) and 95% confidence interval (CI) between the risk of relapse or death and *WWC1* expression. In multivariate analysis, the regression models were adjusted for age at surgery, tumor grade, disease stage, and ER and progesterone receptor (PR) status. To validate the association of *WWC1* expression with breast cancer survival, an online tool named Kaplan–Meier Plotter (http://kmplot.com/analysis/) was used to analyze the relationship between breast cancer survival and *WWC1* expression based on the datasets available online 33. Spearman's rank correlation analysis was performed to identify genes whose expression was correlated with *WWC1* using our microarray data. These *WWC1*‐correlated genes were interrogated by the bioinformatics software Ingenuity Pathway Analysis (IPA), which predicted potential signaling pathways influenced by *WWC1*. *WWC1* methylation data generated by the Illumina HumanMethylation450 BeadChip (HM450) in normal tissue samples in The Cancer Genome Atlas (TCGA) Breast Invasive Carcinoma Provisional Database were downloaded from FireBrowse (http://firebrowse.org). *WWC1* gene methylation and mRNA expression (RNA Seq V2 RSEM) data in TCGA were retrieved from the Web‐based tool cBioPortal (http://www.cbioportal.org/index.do) which only contains methylation data from the probes that had strong negative correlations with methylation signals 34, 35. Correlations between DNA methylation and gene expression of *WWC1* were analyzed using the Spearman correlation coefficient within graphpad prism 7 (GraphPad Software Inc., San Diego, CA, USA). All the statistical tests were performed with the Statistical Analysis System (sas) software, version 9.2 (SAS Institute Inc., Cary, NC, USA). *P*‐values were two‐sided, and those < 0.05 were considered statistically significant.

## Results

### Relationship between *WWC1* expression and patient characteristics

Table 1 shows the clinicopathologic characteristics of 470 breast cancer patients in association with *WWC1* expression measured by qRT‐PCR. Low expression of *WWC1* was significantly associated with advanced‐stage diseases (*P* = 0.018) and high‐grade tumors (*P* < 0.0001). Patients with ER‐negative tumors had lower expression of *WWC1* compared with those with ER‐positive tumors (*P* = 0.0017). A similar relationship was also observed for PR (*P* = 0.00029). A correlation analysis was performed on 204 samples which had *WWC1* expression data generated by both qRT‐PCR and microarray. A significant correlation (Spearman *r* = 0.46, *P* < 0.0001) was found between the two methods. In a TCGA dataset, *WWC1* expression was lower in breast tumors compared to matched adjacent normal tissues (*P* = 0.043; Fig. 3D).

**Table 1:** Associations of *WWC1* expression with clinical and pathological factors of breast cancer.

Variables	Total No. (*N* = 470)	*WWC1* expression			*P*‐value
		Low No. (%)	Mid No. (%)	High No. (%)	
Disease stage					
Stage 1	151 (33.93)	39 (25.83)	50 (33.11)	62 (41.06)	0.018
Stage 2	222 (49.89)	74 (33.33)	80 (36.04)	68 (30.63)	
Stages 3 and 4	72 (16.18)	31 (43.06)	18 (25.00)	23 (31.94)	
Tumor grade					
Grade 1	48 (10.55)	13 (27.08)	10 (20.84)	25 (52.08)	< 0.0001
Grade 2	178 (39.12)	42 (23.60)	71 (39.89)	65 (36.52)	
Grade 3	229 (50.33)	95 (41.48)	73 (31.88)	61 (26.64)	
ER status					
Positive	313 (68.19)	85 (27.16)	114 (36.42)	114 (36.42)	0.0017
Negative	146 (31.81)	64 (43.84)	42 (28.77)	40 (27.40)	
PR status					
Positive	171 (51.82)	43 (25.15)	65 (38.01)	63 (36.84)	0.00029
Negative	159 (48.18)	67 (42.14)	46 (28.93)	46 (28.93)	

### *WWC1* expression and breast cancer survival

**Figure 1:**
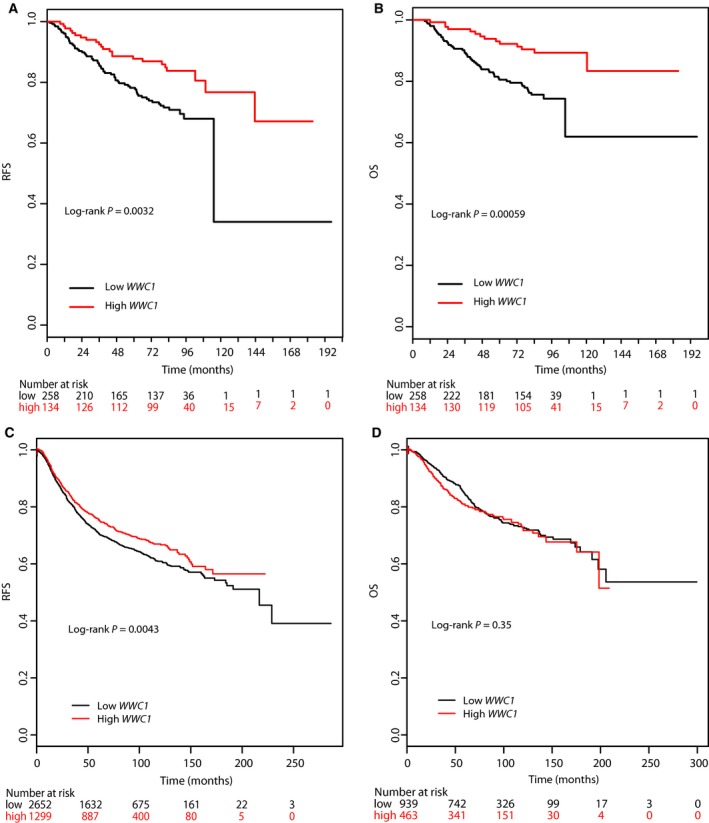
Kaplan–Meier survival curves in patients with high and low *WWC1* expression. (A) RFS curves in patients of our study with high (upper tertile) and low (two lower tertiles) expression of *WWC1*. (B) OS curves in patients of our study with high (upper tertile) and low (two lower tertiles) expression of *WWC1*. (C) RFS curves in patients from online datasets with high (upper tertile) and low (two lower tertiles) expression of *WWC1*. (D) OS in patients from online datasets with high (upper tertile) and low (two lower tertiles) expression of *WWC1*.

Associations of breast cancer survival with *WWC1* expression are shown in Table 2. Compared to those with high expression, patients with low expression of *WWC1* had higher risk of relapse (HR = 1.49, 95% CI: 1.15–1.94, *P* for trend = 0.0028) and higher risk of death (HR = 1.66, 95% CI: 1.23–2.24, *P* for trend = 0.00089). After adjusting for age at surgery, tumor grade, disease stage, and ER and PR status, the association with relapse remained significant (HR = 1.33, 95% CI: 1.003–1.75, *P* for trend = 0.047). Since the associations with survival outcomes were not substantially different between low and medium *WWC1* expression, we grouped the two groups together and compared their disease outcomes with those of high *WWC1* expression. Survival analysis showed that patients with low and mid expression (low and mid tertile) had significantly increased risk of relapse and death (HR = 2.06, *P* = 0.0038, and HR = 2.76, *P* = 0.00095, respectively; Table 2, Fig. 1A,B) compared to those with high expression (high tertile). The association remained significant for relapse after adjusting for clinical and pathological variables (HR = 1.83, *P* = 0.024; Table 2).

**Table 2:** Associations of *WWC1* expression with breast cancer survival.

Variables	RFS		*P*‐value	OS		*P*‐value	RFS^a^		*P*‐value	OS^a^		*P*‐value
	HR	95% CI		HR	95% CI		HR	95% CI		HR	95% CI	
High	1			1			1			1		
Mid	**1.88**	**1.09–3.24**	**0.024**	**2.53**	**1.31–4.87**	**0.0055**	**1.83**	**1.02–3.27**	**0.042**	1.78	0.87**–**3.62	0.11
Low	**2.30**	**1.33–3.99**	**0.0030**	**3.05**	**1.58–5.89**	**0.00093**	**1.83**	**1.02–3.29**	**0.044**	1.94	0.97**–**3.89	0.061
Continuous	**1.49**	**1.15–1.94**	**0.0028**	**1.66**	**1.23–2.24**	**0.00089**	**1.33**	**1.003–1.75**	**0.047**	1.36	0.98**–**1.89	0.068
High (upper tertile)	1			1			1			1		
Low (lower two tertiles)	**2.06**	**1.26–3.37**	**0.0038**	**2.76**	**1.51–5.03**	**0.00095**	**1.83**	**1.08–3.08**	**0.024**	1.86	0.99**–**3.52	0.055

The significant statistical results are indicated in bold.

a Adjusted for age at surgery, tumor grade, disease stage, ER, and PR.

The relationship between breast cancer survival and *WWC1* expression was also analyzed using an online tool, the Kaplan–Meier Plotter, in an online database containing 5134 breast cancer samples with a mean follow‐up of 69 months from 35 publically available microarray datasets (E‐MATB‐365, E‐TABM‐43, GSE11121, GSE12093, GSE12276, GSE1456, GSE16391, GSE16446, GSE16716, GSE17705, GSE17907, GSE18728, GSE19615, GSE20194, GSE20271, GSE2034, GSE20685, GSE20711, GSE51653, GSE2603, GSE16971, GSE2990, GSE31448, GSE31519, GSE32646, GSE3494, GSE37946, GSE41998, GSE42568, GSE45255, GSE4611, GSE5327, GSE6532, GSE7390, and GSE9195). The microarray probe 213085_s_at was used to represent *WWC1* expression which was categorized into high and low groups by upper tertile. As shown in Fig. 1C,D, high *WWC1* expression was associated with RFS (log‐rank *P* = 0.0043), but not with OS.

### *WWC*1‐related pathways

**Figure 2:**
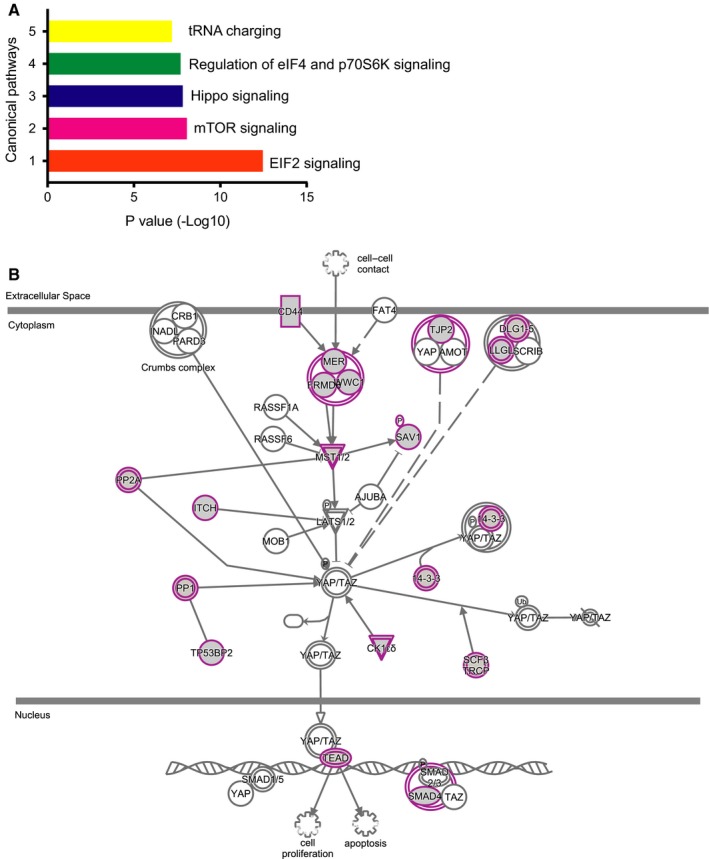
Signaling pathways identified by IPA based on the genes co‐expressed with *WWC1* in our expression microarray data. (A) Top 5 canonical pathways based on *P*‐value; (B) WWC1 in the Hippo signaling pathway. Node shape represents the functional class of gene product. Double outline for complexes/group of different proteins. Purple for upregulation.

To explore the WWC1‐related signaling pathways, we performed co‐expression analysis on genes significantly correlated with *WWC1* expression in our breast cancer microarray data. A total of 3560 out of 22 184 genes were found to be significantly co‐expressed with *WWC1* (FDR *q* value < 0.05 and Spearman correlation coefficient > 0.2). Bioinformatics analysis of these genes by IPA suggested that the top five canonical pathways involved were EIF2 signaling, mTOR signaling, Hippo signaling, regulation of eIF4 and p70S6K signaling, and tRNA charging (Fig. 2A,B).

### *WWC1* methylation status and expression

**Figure 3:**
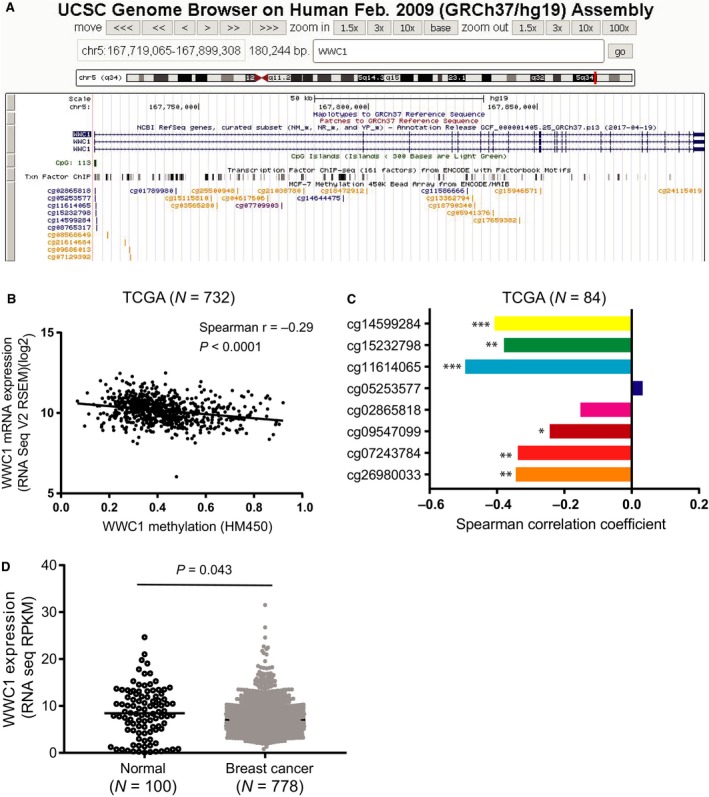
Correlation between *WWC1* expression and methylation in breast cancer in the TCGA database. (A) A screenshot from the UCSC Genome Browser shows the CpG island in the *WWC1* promoter and CpG site probes in the Illumina HumanMethylation450 BeadChip. (B) Scatter plot shows the Spearman correlation between *WWC1* expression and methylation in all CpG sites in the *WWC1* gene using the TCGA breast cancer data from cBioPortal. (C) Bar charts indicate the correlation coefficients between *WWC1* expression and methylation in each of the 8 CpG sites located in the CpG island using data from the TCGA breast cancer and matched normal tissues (Spearman's rank correlation; *P < 0.05; **P < 0.001; ***P < 0.0001). (D) The *WWC1* expression in normal tissue is higher than that in breast cancer using Student's *t*‐test.

Based on the UCSC Genome Browser, a CpG island was identified within the *WWC1* promoter region (Fig. 3A). Eight methylation probes, cg26980033, cg07243784, cg09547099, cg02865818, cg05253577, cg11614065, cg15232798, and cg14599284, were designed in the region by Illumina in the HumanMethylation450 BeadChip. A significant correlation between *WWC1* methylation and expression across all the probes in the gene was observed (*r* = −0.29, *P* < 0.0001; Fig. 3B). Analyzing methylation at each specific probe in relation to gene expression in 84 breast tumor samples and matched adjacent normal tissues, we also found that methylation in most of the probes was inversely correlated with expression (Fig. 3C).

## Discussion

In this study, we found that *WWC1* expression was low in breast tumors compared to normal tissues and that low expression was associated with aggressive tumors and poor RFS. The *WWC1* gene is located in 5q34 with high expression in the brain and kidneys 7. Most studies have investigated the effect of *WWC1* on cognition and memory performance. Although its function in non‐neuronal cells is less clear, emerging evidence suggests that *WWC1* may play a role in cancer. Decreased WWC1 was shown to maintain the stemness of osteosarcoma cells 36. Knockdown *WWC1* expression enhanced the migration and invasion of immortalized breast epithelial cells, and reduced expression of WWC1 in claudin‐low breast cancer was associated with poor prognosis 25. Loss of *WWC1* function could also induce EMT and promote mammary epithelial cell transformation, whereas WWC1 overexpression was found to suppress head and neck squamous cell carcinoma cells in forming colony and oncosphere in soft agar 37. Based on the findings, *WWC1* was considered a potential tumor suppressor 38. A recent study confirmed that WWC1 could inhibit breast tumor metastasis both *in vitro* and *in vivo* 6.

Consistent with the above reports, we found that low expression of *WWC1* was associated with shorter RFS, suggesting that *WWC1* may play a role in the suppression of tumor progression. The association between *WWC1* and RFS was replicated independently in a larger dataset. Despite most studies showing *WWC1* as a possible tumor suppressor, a few studies found inconsistent results. One study showed that high expression of WWC1 was associated with poor prognosis of gastric cancer patients with low expression of atypical protein kinase C 39. A recent study reported that *WWC1* could enhance cell proliferation, migration, and invasion in both immortalized and cancerous prostate epithelial cells, and overexpression of *WWC1* was observed in prostate cancer 14. These inconsistent findings may indicate that WWC1 has tissue‐specific and/or context‐dependent effects on cancer cells. More than 30 public microarray datasets were included in the Kaplan–Meier Plotter analysis, and these datasets had diverse patient background and treatment, which may affect OS outcome.

WW and C2 domain containing 1 was found to regulate growth suppression through the Hippo signaling pathway in *Drosophila* 15, 16, 17, and this finding was confirmed in mammalian cells 40. Based on our microarray data on gene expression, we confirmed that WWC1 was involved in the Hippo signaling pathway in our IPA, which focused on the genes co‐expressed with *WWC1*. We also found that these co‐expressed genes were enriched in the mTOR signaling pathway. Hippo and mTOR are two critical pathways that control cell growth and tissue/organ homeostasis. Previous studies have provided evidence for signaling cross talk between the mTOR and Hippo pathways. YAP, which is the main downstream target of the mammalian Hippo pathway, can regulate the mTOR pathway via its effects on miR‐29 expression 41. *WWC1* could be an important signaling node linking mTOR and Hippo pathways. Promoter methylation that leads to tumor suppressor gene silencing is an important mechanism involved in tumor initiation, progression, and metastasis 42, 43. *WWC1* methylation was found to be associated with unfavorable prognostic indicators in patients with chronic lymphocytic leukemia 44. Epigenetic inactivation of *WWC1* was found frequently in B‐cell acute lymphocytic leukemia 45. One study reported that *WWC1* expression was significantly affected by CpG island methylation in the promoter region and the promoter methylation was elevated in human clear renal cell carcinoma 46. In our study, we found a significant inverse correlation between CpG methylation and *WWC1* expression, suggesting that DNA methylation may regulate *WWC1* expression. We also noticed in our study that methylation regulation was not restricted to the promoter region as methylation of CpG sites in other regions of the gene could be involved (data not shown).

Genetic polymorphisms in the *WWC1* gene may affect expression and contribute to disease risk. One recent study identified *WWC1* as a possible genetic locus for breast cancer susceptibility among women of African ancestry 47. A genomewide association study reported that individuals carrying the T allele of rs17070145 in *WWC1* had significantly better memory scores in the Buschke Selective Reminding Test compared to those carrying the C allele 9. The single nucleotide polymorphism rs17070145 resides in intron 9 of the *WWC1* gene. We found that patients with genotype TT or TC in rs17070145 had better relapse‐free and OS compared to those with genotype CC, but eQTL was not detected in this locus, indicating no impact of this genotype on *WWC1* expression (data not shown). The biological mechanism underlying the genotype's association with breast cancer survival remains to be elucidated.

Of note, the relatively small sample size may limit the statistical power of our study. We also did not evaluate whether our tumor samples had a deletion in chromosome 5q which may explain low expression of *WWC1*.

## Conclusions

In summary, we found that low *WWC1* expression was associated with shorter RFS of breast cancer patients compared to high expression and that the association was independent from tumor grade, disease stage, and hormone receptor status. We further replicated this association in a large independent dataset available online. The study also showed that *WWC1* expression was inversely associated with tumor grade and disease stage and hormone receptor‐positive tumors had higher expression than receptor‐negative ones. These associations indicate that tumor biology and cell differentiation may influence the expression of *WWC1*. Bioinformatics analysis of our microarray data on gene expression confirmed that *WWC1* expression was involved in the Hippo signaling pathway. Analysis of online methylation data indicated that DNA methylation could influence *WWC1* expression. Further investigating the biological actions of WWC1 in breast cancer will help to elucidate the mechanisms of tumor progression.

## Conflict of interest

The authors declare no conflict of interest.

## Author contributions

HY and ZW designed the study, analyzed the data, and wrote the manuscript. ZW, YS, YF, and MT performed the laboratory experiments. HY, ZW, DK, and NB interpreted the data. All authors approved the final version of the manuscript, including the authorship list.
